# A Standardised Vocabulary for Identifying Benthic Biota and Substrata from Underwater Imagery: The CATAMI Classification Scheme

**DOI:** 10.1371/journal.pone.0141039

**Published:** 2015-10-28

**Authors:** Franziska Althaus, Nicole Hill, Renata Ferrari, Luke Edwards, Rachel Przeslawski, Christine H. L. Schönberg, Rick Stuart-Smith, Neville Barrett, Graham Edgar, Jamie Colquhoun, Maggie Tran, Alan Jordan, Tony Rees, Karen Gowlett-Holmes

**Affiliations:** 1 CSIRO Oceans & Atmosphere, Hobart, Tas, Australia; 2 Institute for Marine and Antarctic Studies, University of Tasmania, Hobart, Tas, Australia; 3 School of Biological Sciences & Australian Centre for Field Robotics, The University of Sydney & Sydney Institute of Marine Science, Sydney, NSW, Australia; 4 The Pawsey Supercomputing Centre / WAMSI, Mt Lawley, WA, Australia; 5 Geoscience Australia, National Earth and Marine Observations Group, Canberra, ACT, Australia; 6 The UWA Oceans Institute (M096), Crawley, WA, Australia; 7 Australian Institute of Marine Science, The UWA Oceans Institute (M096), 39 Fairway, Crawley, WA, 6009, Australia; 8 NSW Department of Industries, Nelson Bay, NSW, Australia; 9 Western Australian Museum, Welshpool, Australia; University of Waikato (National Institute of Water and Atmospheric Research), NEW ZEALAND

## Abstract

Imagery collected by still and video cameras is an increasingly important tool for minimal impact, repeatable observations in the marine environment. Data generated from imagery includes identification, annotation and quantification of biological subjects and environmental features within an image. To be long-lived and useful beyond their project-specific initial purpose, and to maximize their utility across studies and disciplines, marine imagery data should use a standardised vocabulary of defined terms. This would enable the compilation of regional, national and/or global data sets from multiple sources, contributing to broad-scale management studies and development of automated annotation algorithms. The classification scheme developed under the Collaborative and Automated Tools for Analysis of Marine Imagery (CATAMI) project provides such a vocabulary. The CATAMI classification scheme introduces Australian-wide acknowledged, standardised terminology for annotating benthic substrates and biota in marine imagery. It combines coarse-level taxonomy and morphology, and is a flexible, hierarchical classification that bridges the gap between habitat/biotope characterisation and taxonomy, acknowledging limitations when describing biological taxa through imagery. It is fully described, documented, and maintained through curated online databases, and can be applied across benthic image collection methods, annotation platforms and scoring methods. Following release in 2013, the CATAMI classification scheme was taken up by a wide variety of users, including government, academia and industry. This rapid acceptance highlights the scheme’s utility and the potential to facilitate broad-scale multidisciplinary studies of marine ecosystems when applied globally. Here we present the CATAMI classification scheme, describe its conception and features, and discuss its utility and the opportunities as well as challenges arising from its use.

## Introduction

Imagery collected by still and video cameras is an effective tool for minimal impact, repeatable observations in the marine environment. Imagery has been used in the marine environment in a scientific context since at least the 1950s [1]. The collection of marine imagery has steadily increased since that time, aided by advances in technology and data storage, and by the increased recognition of the versatility and advantages of this method. Camera systems are particularly useful for collecting visual observations in remote or hazardous environments such as in deep waters beyond safe diving depths, and in areas experiencing extreme tides, high turbidity, ice cover or dangerous marine life (e.g. [2] and references therein). Still and video imagery can be collected from a number of platforms that range in sophistication from diver-held systems, to those towed behind vessels, to cameras deployed on autonomous underwater vehicles (AUVs) and remotely operated vehicles (ROV) [1, 3, 4]. Regardless of the collection platform used, imaging has several advantages over sample collection, although it cannot replace specimen collection for taxonomic work. The advantages include: reducing the time spent retrieving samples from the field and analysing them in the laboratory (although this is balanced by time spent processing imagery); generating a permanent record that can be revisited; an ability to sample a wider range of environments; and, perhaps most importantly, non-destructive sampling, thereby allowing sensitive benthic sites, including those within marine reserves, to be repeatedly sampled with minimal disturbance. Qualitative and quantitative data derived from imagery are used for multiple purposes, such as creating inventories or quantifying the biodiversity and community composition of an area [5–7], describing benthic habitats [8, 9], documenting environmental deterioration due to anthropogenic or natural causes [2, 10–12], interpretation or validation of remotely sensed data [13–16]; establishing relationships for predictive modelling [17–19]; and monitoring for change [3, 20, 21]. Thus, collection and interpretation of imagery has become a standard tool for sampling marine environments.

Because imagery archives represent a permanent record of the environment at a particular point in time and space they will become increasingly valuable given the nature and scale of contemporary issues facing marine systems. While studies that collect and use marine imagery are often local or regional in scale, and annotate imagery with a specific question in mind (e.g. [16, 22]), images and annotations can be re-used or re-analysed, and amalgamated across datasets to address new questions at broader scales. Not only does this maximise the return on investment in collecting and processing imagery, it also allows the generation of amalgamation data sets necessary for state of the environment reporting (e.g. [23, 24]), and for addressing conservation and ecosystem-based questions at the broad scales most relevant to management (e.g. [25, 26]). In an era unprecedented in scale of environmental perturbation [27, 28], and with recent increases in the extent of marine reserves in both coastal and offshore waters [29, 30] new and existing marine imagery will form part of programs that aim to monitor ecological change on regional, or national scales.

Standardised vocabularies of defined terms or ‘labels’ are necessary to enable the amalgamation of local and regional datasets and to realise the full potential of image databases in providing broad-scale and long-term outcomes [31, 32]. In recognition of this, several national or region-wide classification systems have been developed that use marine imagery. These are largely aimed at classifying habitats or biotopes for mapping purposes through a top-down approach—e.g. the European Nature Information System (EUNIS) in Europe [33, 34]; National Intertidal/Subtidal Benthic (NISB) habitat classification scheme in Australia [35]; and the Coastal and Marine Ecological Classification Standard (CMECS) in the United States [36]. These broad classifications rely primarily on semi-quantitative information with respect to substrate types and broad biota classes such as dominant species or community types.

Nevertheless, marine imagery is used for a wide range of purposes and often more detailed information than habitat or biotope type is required. At the finest level of identification, a standardised taxonomic classification exists for marine species through the World Register of Marine Species (WoRMS) [32, 37]. However, even basic taxonomic identification from imagery can be difficult or impossible, and is often not achievable without specimen sampling, expert knowledge, and extensive taxonomic literature, including exhaustive species catalogues or field guides based on local collections [4]. For optimising the use of marine imagery, an intermediary classification vocabulary is required, that conveys as much detail as possible through clearly defined labels, but is flexible enough to be applied across different scales, scoring platforms and techniques, and across images of varying quality. Such classifications have been produced on an *ad hoc* basis by a number of environmental baseline and monitoring programs (government and private) to suit the purpose of their particular program; however, while they identify similar categories the terminology is not consistent (e.g. [38–41]).

We propose that a standardised annotation vocabulary (classification) for identifying taxa, shape and growth forms, and substrates in images would streamline data management, facilitate data sharing and collation for future projects; in addition, it could make historical data more accessible for other users through translations from existing classifications. Furthermore, imagery annotated with consistent, standardised labels could be used as training sets to facilitate the advancement of automated machine-learning approaches to image annotation (e.g. [42–45]); automation of image annotations could lead to significantly improved efficiency and saved time.

To address the issues and needs identified above we developed a flexible, hierarchical classification scheme for annotating physical and biological components observed in imagery through the Collaborative and Automated Tools for Analysis of Marine Imagery (CATAMI) project [46]. Here we introduce the CATAMI Classification Scheme (CCS), and discuss its application potential, utility and limitations.

## Methods

### Expert community and communication

The need for a standard for classifying substrates and biota in marine imagery beyond broad habitat types was identified at an initial stakeholder workshop of the CATAMI Project in March 2012 (S1 Appendix). The CATAMI Classification Scheme (CCS) was pioneered by the CATAMI Technical Working Group (S2 Appendix), a multidisciplinary group of researchers including taxonomic experts, ecologists, and data managers, associated with the majority of Australian research institutions that routinely collect and use marine imagery.

The CATAMI Technical Working Group developed the CCS through video-conference discussions, workshops and e-mails, with refinements based on feedback from interested parties and the wider community during scientific conferences (S3 Appendix) and through on-line blogs. A first draft version of the CCS, documenting each branch of the classification hierarchy with a description and example *in situ* images, was released to the wider Australian scientific community for comment in February/March 2013. Further refinements based on feedback were made prior to the December 2013 release; version 1.4 released in December 2014 contains additional updates [47]. The scheme was further promoted and discussed through national and international workshops and conference presentations (S3 Appendix).

Continued discussions between the members of the CATAMI Technical Working Group and interested parties ensure endurance and longevity of the classification scheme. We welcome feedback regarding the use of the CCS, as well as suggestions for additions to and further refinements of the classification tree. Presently, readers can direct comments and communication to the primary authors (FA, NH, RF and LE).

### The development of the CATAMI Classification scheme (CCS)

Ideally, a classification for benthic substrates and biota in marine imagery should be: (i) applicable across benthic image collection methods (e.g. [1]), annotation platforms [48], and scoring methods (e.g. [49]); and (ii) well described, documented, and maintained.

Existing classifications [33–36] and identification catalogues [38, 50, 51], as well as project-specific schemes for ‘in-house’ use at various institutions were reviewed, and commonalities were identified. Parts of the most detailed existing classifications were adopted into the new scheme, wherever practical (S2 Appendix). In developing a unified scheme with the CCS we aimed at ensuring that it accommodates data collection at varying levels of detail, depending on the needs of different users. A hierarchical structure, with increasingly finer resolution moving through the levels ensures flexibility to accommodate a variety of research questions, image types, and sampling resolutions (from whole of image to individual points), and allows the new classification to dovetail into existing biotope and habitat classifications such as EUNIS [33] or NISB [35].

#### Documentation and maintenance

Clear documentation and description of each branch in the hierarchy is key to wide uptake and longevity of any classification. This was achieved for the CCS through the CATAMI web-site [46] and through the publication of technical documents and reports. To provide a stable, national reference, the CCS classes have furthermore been incorporated into the Commonwealth Scientific and Industrial Research Organisation’s (CSIRO) Codes for Australian Aquatic Biota (CAAB) database [52]. This database represents a curated virtual collection of Australian and Indo-Pacific species and higher taxa. CAAB uses an expanding 8-digit coding system for aquatic organisms and is continuously maintained [52].

## Results

### The CATAMI Classification scheme

**Fig 1:**
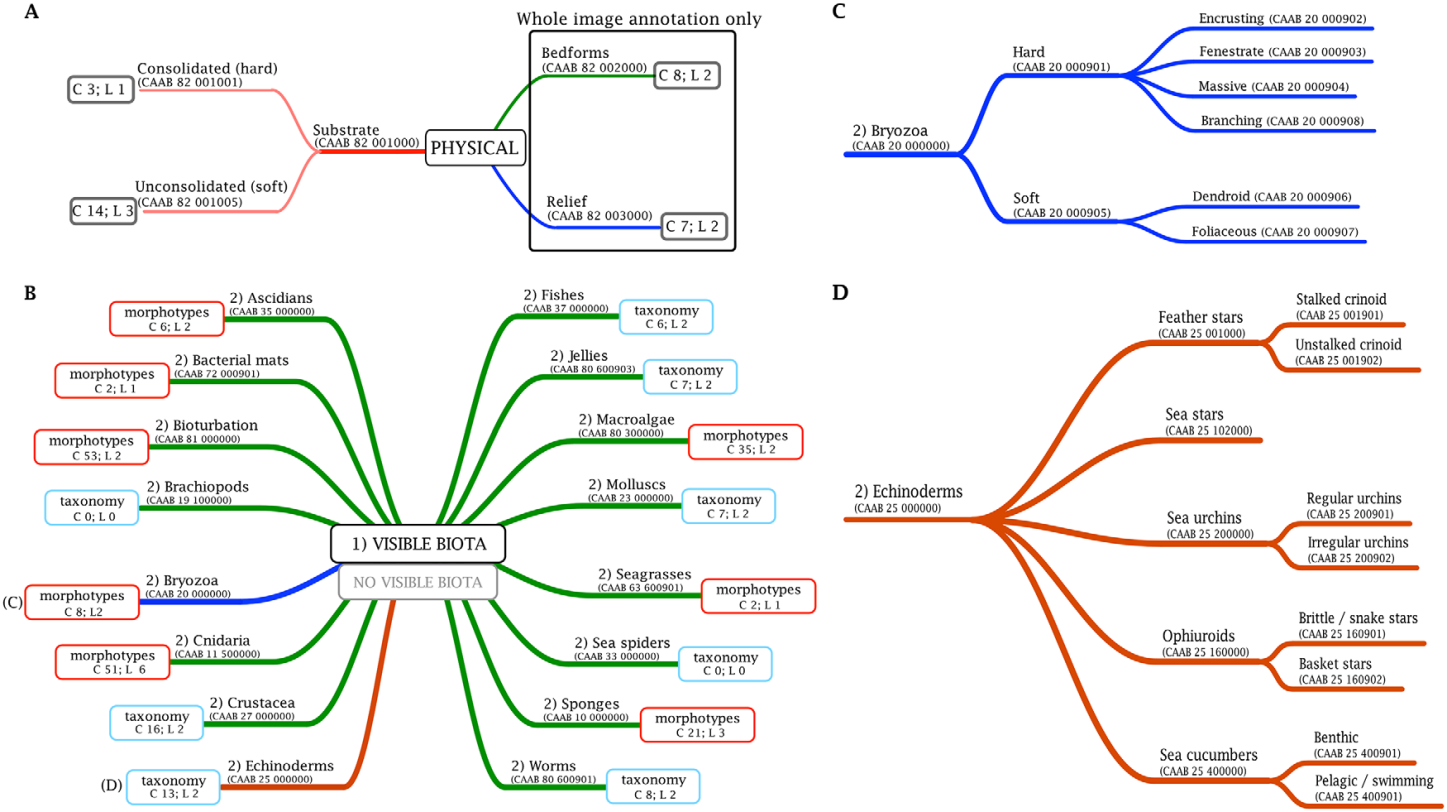
Overview of the coarsest levels of the CATAMI Classification Scheme for (a) physical and (b) biological annotations in imagery; (c) and (d) show details of the ‘bryozoa’ and the ‘echinoderms’ branches. The biological classification at coarsest level is into phyla or broad groups, which are then divided using either taxonomy or morphology (as shown here), depending on what can be more consistently determined from imagery. The number of categories (C) and levels (L) defined under each branch are shown. The full classification scheme can be viewed at http://www.catami.org/classification [53].

The CCS annotates habitats and biota; it was primarily directed towards classification of benthic imagery, but adaptation to pelagic systems is possible through further development of some of the classification branches. The CCS has two main branches, one that describes the physical components of benthic images (36 categories), the other describes the biological components (251 categories) [47]. The biological classification at the coarsest level distinguishes phyla or broad groups, which, subject to the need for resolution, can then be further divided using either taxonomy or morphology (Fig 1), depending on what can be more consistently determined from imagery. The hierarchical structure enables users to record fine-scale detail of morphology (or species) necessary for some studies, but also provides a logical and consistent structure for aggregation of these detailed classes into increasingly coarser groupings, akin to aggregating species into genera or families. The hierarchy also allows consideration of uncertainties in identifications in a consistent way by using coarser levels in the hierarchy. Uncertainties may arise from technical issues such as viewing angle, completeness of object in the frame, image quality or lighting; annotation by non-experts, including the potential use of crowd-sourcing or citizen science, can also warrant the use of coarser levels in the CCS hierarchy. The CCS hierarchy and classes as per date of this publication is illustrated in Fig 1, with the full classification tree available from http://www.catami.org/classification [53].

Additional descriptors such as health status (bleached/unbleached; damaged; etc.), colour or other interpretations can be added to each group by the use of standardised ‘modifiers’[47]. Colour was included as a ‘modifier’ rather than an identifying property, because it is subject to many external factors including biological variability, illumination, distance from the camera or light source, type of light source, light absorption properties of the water, or image post processing just to name a few (e.g. [54]).

The **physical** component of the classification has three branches—substrate, relief and bedform (Fig 1a). **Substrate** refers to the types of bottom material that are visible in the scoring area. This group has two coarse subdivisions, unconsolidated (i.e. soft substrates) and consolidated (i.e. hard substrates) (Fig 1a). Finer-level classification considers assessment of grain size (e.g. pebble/gravel, 2–10 mm) [47, 53]. Modifiers for substrate types include, for example, ‘veneer’, which applies to rock beneath a thin sediment layer as indicated by the presence of attached sessile biota, although only unconsolidated sediment may be visible in the image. **Relief** describes the height and structural complexity of the substrate [47, 53]. **Bedform** (e.g. sandwaves and ripples) refers to features caused by the transport of unconsolidated sediment over the seabed as the result of water movement or animal activity. The CCS categorizes bedforms based on height and dimensionality [47, 53]. Relief and bedform can only be identified across a whole image or transect, because they represent broad-scale features than cannot be captured by a single point within an image.

The **biological** classification at its coarsest level considers the presence or absence of any visible biota or traces thereof (bioturbation) (Fig 1b). The next level corresponds to a major biological group, usually phylum, although in some cases where organisms are often small and difficult to distinguish, phyla are combined (e.g. ‘worms’ refers to a series of worm-like phyla including annelids, sipuncula, echiura; ‘jellies’ represent gelatinous biota including medusae, salps, etc. [47, 53]). Bioturbation–visible traces of biota [39]–is added as a separate group of biota at this level (Fig 1b). Subsequent levels (i.e. 3^rd^ tier and finer) include coarse-level taxonomic classification (phylum, order, class) and morphology (shape, growth form), depending on which system was most sensible to use for imagery (Fig 1b; [47, 53]). For example, identification of sponges, octocorals or stony corals, even to the level of family or genera, relies on microscopic examination of spicules, sclerites or corallites; in addition, a single sponge species can show significant morphological plasticity dependent on environmental conditions (e.g. [55–59]). In these cases the use of growth forms provides a more consistent classification, and avoids pitfalls and errors that are common when attempting detailed taxonomic classification from imagery. Furthermore, it entails more information regarding function, ecology and selective forces of environmental factors than phylum-level taxonomy alone [60].

**Fig 2:**
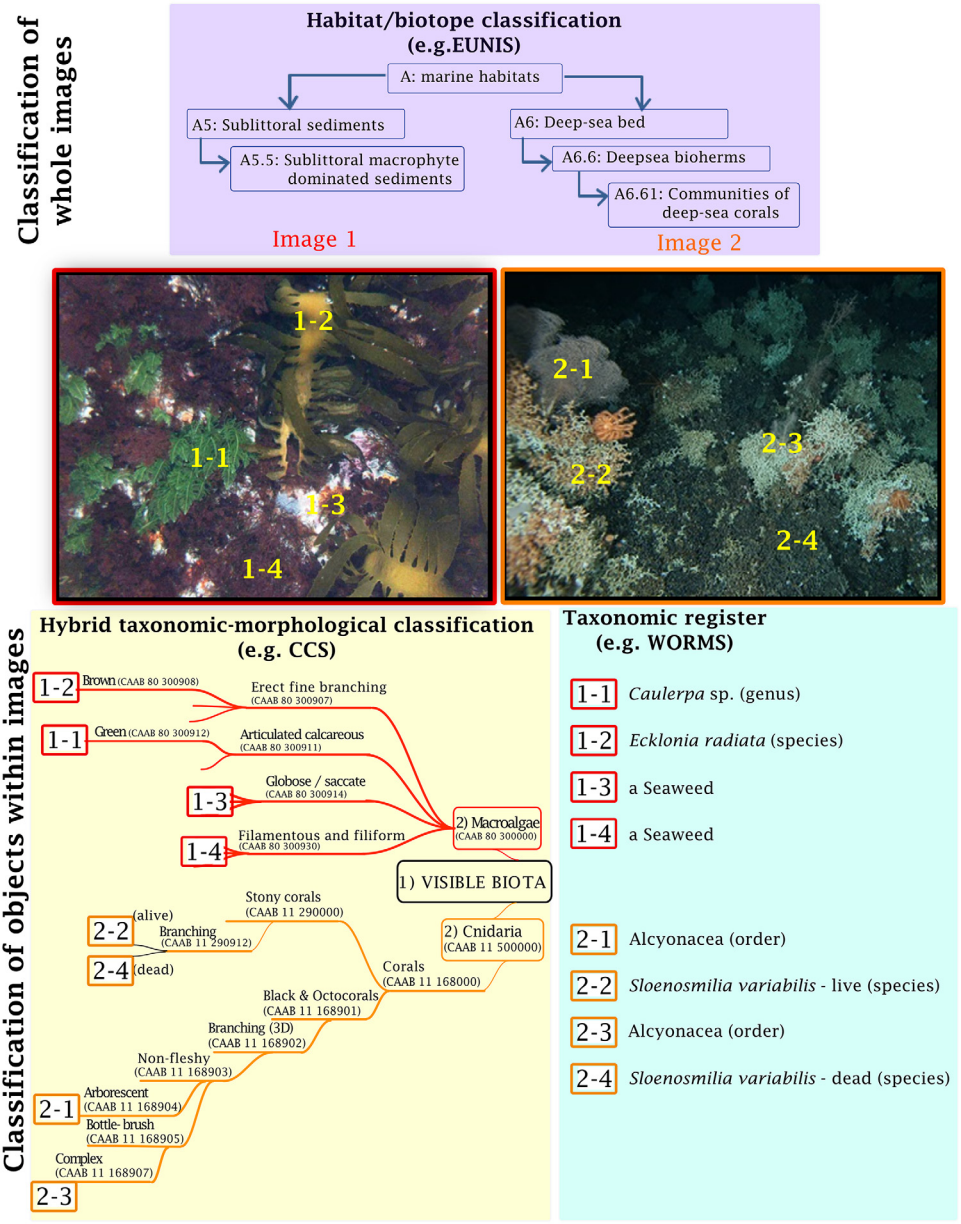
Comparison of the classification of two underwater images using a habitat/biotope classification (EUNIS–[33, 34]), the CATAMI Classification Scheme (CCS) and a taxonomic classification (WoRMS–[37]). The figure illustrates the level of detail achieved within each classification system. Image 1: taken by IMAS with the ACFR AUV off the east coast of Tasmania, Australia at 24 m depth; Image 2 taken by CSIRO with the ‘Deep Camera Platform’ at Hill U Seamount south of Tasmania, Australia at 1167 m depth.

With the exceptions of relief and bedforms, the CCS classes can be applied to individuals or scoring points within images (Fig 2), they can also be combined qualitatively or quantitatively to describe biological communities or biotopes that form the finest level classes in existing standardised habitat classification schemes (*sensu* [33, 35, 36]). The ability to annotate features or individuals at a point in a given image is essential, as the most common scoring methods used for imagery rely on this method for quantitative estimates or measurements of relative abundance of substrate or biota types (see [49]). Percentages of different biota or substrate types are usually based on a number of point measurements within a known area or field of view (e.g. using Coral Point Count, [61, 62]).

#### Documentation and maintenance

Detailed descriptions of all levels of the CCS are accessible at http://catami.org/classification in three documents: (1) CATAMI Technical Working Group (2013) [47] that describes the CCS; (2) a visual guide including example images of the various classes [47]; and (3) the CATAMI Code file that contains the CCS hierarchy in a tabulated format that can be imported to annotation software. Respective unique 8-digit CAAB identification codes allocated to each of the CCS classification levels can be accessed through http://www.cmar.csiro.au/caab/ [52]. Most researchers in Australia are familiar with the CAAB system and use it for species identification in databases, as it helps avoid errors inherent in text labels and allows automated updating as taxa are revised. Protocols are being developed for proposing the addition of new branches or potential changes to the CCS. Any accepted additions or changes will be documented and disseminated through the CATAMI and CAAB websites [46, 52].

#### Uptake

The CATAMI Classification scheme (CCS) is now in use since 2013 and has been taken up by numerous local, national and international users. It has been adopted across Australia’s marine community involved in ongoing processing of marine imagery, including government organisations, academic institutions and private industry. As of April 2015, 784 copies of the visual guide, 503 copies of the technical document and 358 copies of the code file have been downloaded from the CATAMI CCS website [46], indicating wide interest in the scheme. New image data collected under Australian national and regional marine monitoring programs are annotated using the CCS (e.g. National Environmental Research Program [63]; New South Wales marine parks [64]). Consultants contracting to oil and gas and other resource companies are also adopting the CCS for project, whereas before this capacity has been lacking within the industry (Ben Brayford, pers. comm. 19th May 2014). In addition the CCS is incorporated into image processing protocols [65] and monitoring strategies [66, 67]. Beyond Australia, benthic images taken as a routine component of surveys undertaken in the Reef Life Survey program (RLS; [68]), are now also scored using the CCS. This has generated a substantial degree of international standardisation—the RLS photo-quadrat database contains ~100,000 images associated with >7,000 reef transects surveyed across 82 ecoregions globally. Presently, the CCS is mainly employed for approaches using underwater imagery, but it has been suggested that diver-conducted work can also benefit from the CCS, e.g. to study low light habitats such as Mediterranean caves or when assessments are done by non-experts [69].

The CCS is also the classification scheme underlying two web applications being developed for image scoring—CATAMI of the CATAMI Project [46] and Squidle of the Australian Centre for Field Robotics (ACFR), University of Sydney [70]. In addition, the CCS labels have been added to the annotation labels available for use in CoralNet [71]. The use of the CCS will ensure data compatibility across all the different approaches and between users.

## Discussion

### The CATAMI Classification scheme

The rapid acceptance and the success of the CCS are likely based on the wide scope of application paired with its versatility, as well as its documentation and curation [46, 47, 52, 53]. The CCS is easy to implement across all collection methods and tools, geographic extents, environments and ecosystems. Combining morphology with high-level taxonomy allows the recording of detail regarding functionality and structure that coarser-level taxonomy alone cannot convey. For example, communities of erect branching corals or sponges often present different habitats and environments than low, encrusting forms of the same taxa (e.g. [72]). In addition, morphologies can be compared between vastly different systems such as climate zones, provincial regions or depths. The CCS is aimed at broad uptake across users from the scientific community, universities, industry, and government departments. It is particularly designed to suit an intermediate level of taxonomic expertise where classification is required across many taxonomic groups. Detailed taxonomic species lists are not necessary for annotating imagery using the CCS, allowing annotation of imagery to individually defined morphotypes in the CCS, where no taxonomic references such as field guides or comprehensive species collections (identified by taxonomists) are available. However, where the taxonomy is known it can be included as additional levels within or in parallel to the CCS, thus recording functional morphology traits as well as species, genera or even family-level information, and retaining the ability to aggregate data at coarser levels within the classification hierarchy for comparison with other regions.

The hierarchical structure is particularly useful when data from studies with different foci are combined for 'higher' level comparison of data across broad regions. Similar to global analyses of collated specimen records at family- or class-level (e.g. [73]).

The CCS is not region specific, and thus it has the potential to be adopted for image annotation worldwide. Taxonomic data for well-known species from collections are necessary to identify bioregions (e.g. [74]) and bathomes (e.g. [75]), but nested within those are biotopes and habitats ([34–36]) that can be described and typified using the CCS, without the need for detailed taxonomic knowledge of the biota (Fig 2). While it is difficult to anticipate future goals associated with annotation of marine data, the CCS is designed to increase flexibility and detail in image annotation, bridging the gap between habitat descriptors and species records.

### Utility

The CCS provides a framework for marine image annotation that fills a critical gap between coarse, habitat-level classifications and purely taxonomic classifications. Because it is standardised it represents a significant improvement on *ad hoc* or agency-specific scoring schemes and has enormous potential for facilitating research and habitat identification from a range of perspectives. Standardised annotation categories create the opportunity to collate and combine historical, contemporary and future datasets from different sources to answer a range of questions across larger spatial and temporal extents than possible with individual datasets. They also facilitate the delivery of standardised datasets to national and international repositories. In addition, the CCS has applications to both region-specific and broad-scale monitoring initiatives, can provide guidance and streamline new annotation initiatives, and enables increased efficiency in annotating imagery via facilitating citizen scientist projects and the development of computer vision algorithms.

#### Increased opportunities for collaboration, data sharing, and answering the ‘big’ questions

Combining datasets increases the quantity and coverage of data that can be used for answering ecological and/or management questions at the broad scales that will increasingly be needed to tackle contemporary issues. Many regional or global models draw on data collated from collection records and published surveys with comparable taxonomic groups classified to a common level such as species, or class ([73, 76, 77]). Without a common vocabulary this is not easily achieved. For example, Beijbom et al. [42] needed to construct a ‘consensus-label set’ before using image annotation data from four different studies. Similarly, in this article we have cited over 20 Australian publications using data collected from imagery. Despite many commonalities in the ‘in house’ developed classification schemes underlying these data, there is little congruence in the terminology used. Thus, the data are not easily compared or amalgamated into a national data set. This is currently being addressed with many Australian organisations translating their historical image data into the CCS. When combined with the uptake of the CCS by ongoing research projects (which avoids the need for translation) a wealth of standardised imagery data will soon be available across a range of biomes to begin addressing some of these broad-scale questions.

#### Facilitation of data delivery

Funding bodies increasingly demand that data generated through public funding become publicly available so they will be discoverable and available for wider use (e.g. Integrated Marine Ocean Observing System [78]; Australian Marine National Facility [79]). Similarly, many international journals–e.g. Nature, PLoS ONE, Ecological Applications–require publication of the data underlying analyses. The most effective way for publishing data is through major national data infrastructure such as the Australian Ocean Data Network web-portal (AODN [80]) and Australian National Data Service (ANDS [81]) in Australia, from where the data can be harvested into global data portals such as the Ocean Geographic Information System (OBIS [82]), the SERPENT Project [83] or PANGEA [84]. It is desirable that such data are comparable between studies, thus that they are reported with standardised categories that are widely used and understood; in addition, clear description of the data collection methods are essential for publication. Ultimately, having standardised terminology in image annotations will maximize publishing opportunities, which will increase discoverability, access and thus efficiency, both by capitalising on existing observational data and by facilitating the assembly of a more cohesive and contextual broad-scale picture of marine habitats..

#### Application to monitoring

Indicator species or taxa have been proposed as an effective tool for assessing ecosystem health and monitoring change [85, 86], with long-lived sessile species such as macroalgae, corals and sponges expected to convey the most relevant responses (e.g. [87]). In coastal ecosystems macroalgae are known to respond to anthropogenic pressures, and a range of indices based on macroalgae have been proposed for international assessment legislation such as the European Union’s Water Framework Directive (WFD, 2000/60/EC) and Marine Strategy (MSFD, 2008/56/EC). All indices are based on the concept that under increasing anthropogenic pressure perennial macroalgae species are typically replaced by opportunistic species, and that the overall richness and cover often declines. The Ecological Evaluation Index (EEI; [88]), for example, categorises species based on their morphological and functional forms, using Littler & Littler [89], into late successional species (ecological state group I) and opportunistic species (ecological state group II). The macroalgae branch of the CCS primarily builds on concepts and morphotypes in Littler & Littler [89], thus it can be readily aligned with the EEI and other macroalgal indicator categories (see Table 1 as an example).

**Table 1:** CATAMI macroalgal classification aligned with life-history characteristics often used in the derivation of indicators, and with the ecological status groups for monitoring the health of marine systems.

CATAMI Level 3 Category	CATAMI Level 4 Category	Description	Successional Status	Opportu-nistic?	Ecol. State Group	Examples of CATAMI group
Filamentous / filiform	Green, Red, Brown	Appears very fine and thread- or hair-like but may not necessarily be technically a filament	Early successional	Yes	II	*Chaetomorpha*, *Polysiphonia*, *Ceramium*, *Ectocarpus*
Sheet-like / membraneous	Red, Brown	Thin, delicate and often translucent. A flattened and sheet-like structure	Early successional	Some	II	*Kallymenia*, *Dictyota*
Sheet-like / membraneous	Green	As above	Early successional	Yes	II	*Enteromorpha*
Globose / saccate	Green, Red, Brown	Spherical shape or balloon-like form.	Early successional	No	II	*Colpomenia*, *Leathesia*
Laminate	Green, Red, Brown	Low profile, plate-like and lobed forms	Mid- successional	No	I/II	*Peyssonnelia*, *Padina*, *Lobophora*
Erect fine branching	Green, Red, Brown	Distinct branching form with a vertical growth habit. Branches are small or narrow	Mid- successional	No	II	*Gracilaria*, many *Caulerpa*, *Lobospira*
Erect coarse branching	Green, Red, Brown	Distinct branching form with a vertical growth habit. Branches are robust or have broader blades than fine-branching	Late- successional	No	I	*Sargassum*, *Cystophora*, many *Codium*
Large canopy-forming	Brown	Large (>>50 cm when mature) and robust, habitat- forming species. Generally large and distinctive fucoids and kelps	Late- successional	No	I	*Ecklonia*, *Phyllospora*, *Laminaria*
Articulated calcareous	Green, Red, Brown	Jointed or segmented, calcified algae	Late- successional	No	I	*Amphiroa*, *Corallina*, *Halimeda*
Encrusting	Red, Brown	Crust-like; thin form growing flattened and closely adhering to the substratum.	Late- successional	No	I	crustose coralline reds, *Ralfsia*

A range of macroalgal indices have been proposed for subtidal environments that utilise morphological and biological traits of species or groups. Table 1 shows how CATAMI macroalgal classifications aligned with life-history characteristics often used in the derivation of indicators, and with the ecological status groups proposed by Orfandis et al. for monitoring the health of marine systems [88].Ecological state group I—late successional species, ecological state group II—opportunistic species.

Other branches of the CCS hierarchy have similar application potential. The reporting of tropical reef health status is generally based on the composition of morpho-functional groups of stony corals (e.g. [90–93]), and vulnerable marine ecosystems (VME) in deeper environments are characterised by habitat forming, erect epifauna such as corals and sponges (e.g. [94–96]). Macroalgal EEI, reef health and VME classification are all based on coarse taxonomy combined with morphological characteristics and thus can be readily identified using the CCS hierarchy.

#### Facilitating the development of protocols and standards for image annotation

The CCS provides a framework for identifying and labelling structures in marine images, without being prescriptive regarding methods for the collection and scoring of marine imagery. It can, nevertheless, guide and streamline the process of developing protocols when setting up new studies. It can also facilitate discussions between research providers and clients regarding the level of detail and outcomes required or achievable, when planning observational studies. In addition, enabling assessment across a wide spectrum of taxa through the CCS can bring certain groups into focus that otherwise may be overlooked. For example, sponges are often only scored generically as ‘sponges’ or ‘filter feeders’, despite being important and diverse components of benthic ecosystems (e.g. [72] and references therein). The sponge classification scheme developed by Schönberg and Fromont [60] and adopted in the CCS now enables scoring of this phylum to a degree that allows at least basic ecologic interpretation of resulting data, and their meaningful inclusion in environmental assessment and monitoring [97].

With a standard vocabulary, the foundation is laid for developing protocols or ‘standards’ for image processing. National or international standards document *‘requirements*, *specifications*, *guidelines or characteristics that can be used consistently to ensure that materials*, *products*, *processes and services are fit for their purpose*’ [98]. These standards can then be referred to in legislation and guidelines. For example the international association of Oil and Gas Producers refers to a series of standards to specify the required sampling methods (none using imagery) for offshore environmental monitoring by the oil and gas industry in the United Kingdom [99]. In Australia, the provision of environmental impact statements is a State and Federal Government condition for development approvals in resource projects such as oil and gas mining, as outlined in the guidelines from the National Offshore Petroleum Safety and Environmental Management Authority [100]. However, these guidelines do not specify standardised metrics or sampling methods for such assessments. The CCS provides the classification that could be used for developing a ‘standard’ for environmental assessments or monitoring based on non-extractive, observational data.

#### Efficient and novel approaches to image scoring

Annotating large volumes of marine imagery collected by still and video platforms is a time consuming process and a standardised classification scheme opens exciting opportunities to increase processing efficiency. Automatic image classification and annotation of marine imagery using computer vision algorithms has rapidly advanced in recent years [42, 101]. Automation algorithms can be divided into supervised and unsupervised ones. Unsupervised algorithms are not capable of identifying broad classes of benthic organisms consistently with high accuracy; therefore they are most useful for classification at the coarsest levels of the CCS (e.g. sand *vs*. algae). On the other hand, supervised algorithms can achieve accuracy levels that are similar to those of human scorers [43, 45]. However, large amounts of training data are needed for supervised algorithms to achieve target accuracies, especially when the goal is to generate algorithms that can classify images across a range of different sampling sites and conditions. Critically, these large volumes of training data must be scored consistently to be of use (e.g. [42]). The CCS provides these consistent labels within a hierarchical classification scheme and as such has the potential to make a significant and unprecedented contribution to the field of computer vision and supervised classification algorithms for underwater benthic imagery. The ultimate outcome of better classification algorithms will be reducing processing costs and the lag time between data collection, statistical analysis and ecological or management applications.

The engagement of citizen scientists, enthusiastic non-experts who complete tasks that contribute to scientific programs, presents another opportunity to increase the efficiency of both capturing *in situ* images and image annotation. Engaging enthusiastic non-experts enables field data capture to occur over much larger spatial and temporal scales than has previously been possible (e.g. [102–104]) and several projects in Australia currently utilise citizen scientists for collecting marine imagery (e.g. [68]). Likewise, the work of citizen scientists could be harnessed for annotating imagery, a process also known as crowd sourcing. In fact, crowd sourcing has already been effectively used to score marine imagery for either very broad categories (such as fish, sand etc. [105]) or specific organisms (e.g. kelp [106]). The range of organisms considered with crowd sourcing could be expanded using standardised CCS categories that are clearly described, and with CCS-based public-access databases, such as CATAMI [46] and Squidle [70]. The increased capacity offered by citizen scientists armed with digital cameras, the internet and a standardised scoring system offers enormous potential for increasing our understanding of marine ecosystems and their status of health.

### Challenges

The CCS facilitates collation and combination of datasets across large geographical and bathymetric scales, maximizing the potential value of the data. However, combining datasets collected for a range of different purposes presents a number of pragmatic, logistic and analytical challenges (e.g. [107]). While the CCS provides a framework for labelling the physical and biological components of marine imagery, it does not intend to provide standardised methods for the collection of marine imagery or the scoring approach used (e.g. whole-image viewing *vs*. point-scoring; [2]). Nor does it prescribe to what level within the CCS an analyst must score. These decisions are still based on the specific purpose of individual projects, and need to be clearly documented in metadata accompanying any published data set (e.g. [54]). Inevitably, combining multiple datasets will result in some loss of resolution and/or information. For example, detectability of taxa may be inherently different between the different sampling platforms (e.g. high resolution still images *vs*. video imagery), while sampling priorities and scoring effort can also differ between surveys using similar platforms. From our own experience, it is difficult to combine data across several platforms and scoring methods, and in the worst case scenario we can be left with presence-only data at a coarse level in the classification hierarchy. However, this is not a challenge unique to image data, large-scale distribution data from sources such as museum records, online databases and citizen science programs usually represent presence-only data and can be at coarse taxonomic resolution (e.g. [73, 107]. The increased desire to analyse and interpret large-scale data sets has prompted an active area of research into the development of new methods to analyse presence–only data [108–110], or combined data with different response variables (e.g. presence-only, presence-absence, abundance; e.g. [111]). In addition, mixed effects and hierarchical models that can capture some of the bias of the data collection methods are likely to be a useful approach [107]. Ultimately, pragmatic and analytical decisions on combining datasets will need to be made based on the focus of individual research questions.

## Conclusion

Imagery and associated derived data are increasingly important tools for minimal impact, repeatable observations in the marine environment. In addition, an increasing need exists to publish data to ensure their longevity, with increasing reliance on digitally accessible data for large-scale studies, and modelling. The CCS caters to the need for standardised, defined terminology which is fundamental for broad dissemination and uptake of data. The CCS vocabulary can be used to classify physical features and biota in order to describe and quantify (statistically or otherwise) biological communities or habitats in imagery. The scheme is collaboratively designed to be easily accessible, adaptable, and agile, with the potential to translate existing data into the scheme. The strength of the CCS lies in its ability to encompass multiple scales and resolutions, with flexibility that allows its use with most scoring methods and annotation platform types for underwater imagery and video. Longevity of the CCS is enhanced by continual maintenance and curation through the CAAB coding system by CSIRO [52]. By sharing, adapting and demonstrating the use of the CCS through various projects across Australia, we continually evolve and keep pace with global trends in innovation through review, use and uptake by the scientific community. As demonstrated, the CCS scheme is already widely implemented, not only in academia, but also in industry and governmental departments across Australia. Because the CCS is not region specific, it has the potential to be adopted globally, or at least to contribute to a global approach, which is currently lacking but sorely needed. By facilitating access to image annotations for scientists and the public through providing an online framework of a nationally accepted protocol for labelling marine imagery, not only do we build on the work conducted in earlier research programs, we enable others, in turn, to build upon our own.

## Supporting Information

S1 Appendix CATAMI Project: Initial stakeholder workshop—aims, participants & outcomes. (DOCX)

S2 Appendix List of the 19 members of the CATAMI Technical Working Group and additional contributors to the CATAMI classification scheme (CCS). (DOCX)

S3 Appendix Meetings where the CATAMI Classification was presented and discussed. (DOCX)

## References

[1] Mallet D, Pelletier D. Underwater video techniques for observing coastal marine biodiversity: A review of sixty years of publications (1952–2012). Fish Res. 2014;154:44–62. 10.1016/j.fishres.2014.01.019 WOS:000336117000006.

[2] Lafratta A, Fromont J, Speare P, Schönberg CHL. Coral bleaching in northwestern Australia. Biol Bull. in review.

[3] Williams SB, Pizarro OR, Jakuba MV, Johnson CR, Barrett NS, Babcock RC, et al Monitoring of benthic reference sites using an autonomous underwater vehicle. IEEE Robot Autom Mag. 2012;19(1):73–84. 10.1109/mra.2011.2181772 WOS:000302539600013.

[4] Howell KL, Bullimore RD, Foster NL. Quality assurance in the identification of deep-sea taxa from video and image analysis: response to Henry and Roberts. ICES J Mar Sci. 2014;Advance Access April 9 2014. 10.1093/icesjms/fsu052

[5] Buhl-Mortensen L, Buhl-Mortensen P, Dolan MFJ, Dannheim J, Bellec V, Holte B. Habitat complexity and bottom fauna composition at different scales on the continental shelf and slope of northern Norway. Hydrobiologia. 2012;685(1):191–219. 10.1007/s10750-011-0988-6 WOS:000300673500013.

[6] Spencer ML, Stoner AW, Ryer CH, Munk JE. A towed camera sled for estimating abundance of juvenile flatfishes and habitat characteristics: Comparison with beam trawls and divers. Estuar Coast Shelf Sci. 2005;64(2–3):497–503. 10.1016/j.ecss.2005.03.012 WOS:000230873200034.

[7] Thresher RE, Althaus F, Adkins J, Gowlett-Holmes K, Alderslade P, Dowdney J, et al Strong depth-related zonation of megabenthos on a rocky continental margin (~700–4000 m) off southern Tasmania, Australia. PLoS ONE. 2014;9(1):e85872 10.1371/journal.pone.0085872 24465758 PMC3899097

[8] Barrett NS, Edgar GJ. Distribution of benthic communities in the fjord-like Bathurst Channel ecosystem, south-western Tasmania, a globally anomalous estuarine protected area. Aquat Conserv-Mar Freshw Ecosyst. 2010;20(4):397–406. 10.1002/aqc.1085 WOS:000279822100005.

[9] Kloser RJ, Williams A, Butler A. Exploratory Surveys of Seabed Habitats in Australia’s Deep Ocean using Remote Sensing—Needs and Realities In: Todd BJ, Greene HG, editors. Mapping the seafloor for habitat characterization: Geological Association of Canada, Special Paper Geological Association of Canada, Special Paper. 47 2007 p. 93–110.

[10] Althaus F, Williams A, Schlacher TA, Kloser RK, Green MA, Barker BA, et al Impacts of bottom trawling on deep-coral ecosystems of seamounts are long-lasting. Mar Ecol-Prog Ser. 2009;397:279–94.

[11] Langlois TJ, Harvey ES, Meeuwig JJ. Strong direct and inconsistent indirect effects of fishing found using stereo-video: Testing indicators from fisheries closures. Ecol Indic. 2012;23:524–34. 10.1016/j.ecolind.2012.04.030 WOS:000307130300055.

[12] Williams A, Schlacher TA, Rowden AA, Althaus F, Clark MR, Bowden DA, et al Seamount megabenthic assemblages fail to recover from trawling impacts. Mar Ecol. 2010;31:183–99.

[13] Kloser RJ, Penrose JD, Butler AJ. Multi-beam backscatter measurements used to infer seabed habitats. Cont Shelf Res. 2010;30(16):1772–82. 10.1016/j.csr.2010.08.004 WOS:000283205300007.

[14] Lucieer V, Hill NA, Barrett NS, Nichol S. Do marine substrates 'look' and 'sound' the same? Supervised classification of multibeam acoustic data using autonomous underwater vehicle images. Estuar Coast Shelf Sci. 2013;117:94–106. 10.1016/j.ecss.2012.11.001 WOS:000315323600010.

[15] Rooper CN, Zimmermann M. A bottom-up methodology for integrating underwater video and acoustic mapping for seafloor substrate classification. Cont Shelf Res. 2007;27(7):947–57. 10.1016/j.csr.2006.12.006 WOS:000246058100005.

[16] Siwabessy PJW, Daniell J, Li J, Huang Z, Heap AD, Nichol S, et al Methodologies for seabed substrate characterisation using multibeam bathymetry, backscatter and video data: A case study from the carbonate banks of the Timor Sea, Northern Australia. Canberra, Australia: Geoscience Australia, 2013 Contract No.: Record 2013/011.

[17] Foster SD, Bravington MV, Williams A, Althaus F, Laslett GM, Kloser RJ. Analysis and prediction of faunal distributions from video and multi-beam sonar data using Markov models. Environmetrics. 2009;20:541–60.

[18] Hill NA, Lucieer V, Barrett NS, Anderson TJ, Williams SB. Filling the gaps: Predicting the distribution of temperate reef biota using high resolution biological and acoustic data. Estuar Coast Shelf Sci. 2014;147:137–47. 10.1016/j.ecss.2014.05.019 WOS:000341477000014.

[19] Hill NA, Pepper AR, Puotinen ML, Hughes MG, Edgar GJ, Barrett NS, et al Quantifying wave exposure in shallow temperate reef systems: applicability of fetch models for predicting algal biodiversity. Mar Ecol-Prog Ser. 2010;417:83–U100. 10.3354/meps08815 WOS:000284006800008.

[20] Bridge TCL, Ferrari R, Bryson M, Hovey RK, Figueira WF, Williams SB, et al Variable Responses of Benthic Communities to Anomalously Warm Sea Temperatures on a High-Latitude Coral Reef. PloS ONE. 2014;9(11): e113079 10.1371/journal.pone.0113079 25426718 PMC4245080

[21] Ferrari R, Gonzalez-Rivero M, Ortiz JC, Mumby PJ. Interaction of herbivory and seasonality on the dynamics of Caribbean macroalgae. Coral Reefs. 2012;31(3):683–92. 10.1007/s00338-012-0889-9 WOS:000307287400007.

[22] Althaus F, Williams A, Kloser RJ, Seiler J, Bax NJ. Evaluating geomorphic features as surrogates for benthic biodiversity on Australia's western continental margin In: Harris PT, Baker EK, editors. Seafloor geomorphology as benthic habitat—GeoHab Atlas of seafloor geomorphic features and benthic habitats. Waltham, MA, USA: Elsevier Insights; 2012 p. 665–79.

[23] State of the Environment 2011 Committee. Australia state of the environment 2011—Independent report to the Australian Government Minister for Sustainability, Environment, Water, Population and Communities. Canberra, Australia: 2011.

[24] Zann LP. State of the marine environment report for Australia: technical summary. Cabberra, Australia: Rescue 2000 & Great Barrier Reef Marine Park Authority, Department of the Environment, Port and Territories, 1996.

[25] Molnar JL, Gamboa RL, Revenga C, Spalding MD. Assessing the global threat of invasive species to marine biodiversity. Front Ecol Environ. 2008;6(9):485–92. 10.1890/070064 WOS:000260800800019.

[26] Przeslawski R, Daniell J, Anderson T, Barrie JV, Heap A, Hughes M, et al Seabed habitats and hazards of the Joseph Bonaparte Gulf and Timor Sea, Northern Australia. Canberra, Australia: Geoscience Australia, 2011 Contract No.: Record 2011/040.

[27] Guinotte JM, Fabry VJ. Ocean acidification and its potential effects on marine ecosystems. Ann N Y Acad Sci. 2008;1134:320–42. 10.1196/annals.1439.013 . 18566099

[28] Lough JM. 1997–98: Unprecedented thermal stress to coral reefs?. Geophys Res Lett. 2000;27:3901–4. 10.1029/2000GL011715

[29] Devillers R, Pressey RL, Grech A, Kittinger JN, Edgar GJ, Ward T, et al Reinventing residual reserves in the sea: Are we favouring ease of establishment over need for protection? Aquat Conserv: Mar Freshwat Ecosyst. 2014.

[30] Spalding MD, McIvor AL, Beck MW, Koch EW, Moller I, Reed DJ, et al Coastal ecosystems: A critical element of risk reduction. Conserv Lett. 2014;7(3):293–301. 10.1111/conl.12074 WOS:000337590000018.

[31] Costello MJ. Distinguishing marine habitat classification concepts for ecological data management. Mar Ecol-Prog Ser. 2009;397:253–68. 10.3354/meps08317 WOS:000273968400024.

[32] Costello MJ, Bouchet P, Boxshall G, Fauchald K, Gordon D, Hoeksema BW, et al Global coordination and standardisation in marine biodiversity through the World Register of Marine Species (WoRMS) and related databases. PloS ONE. 2013;8(1):e51629 10.1371/journal.pone.0051629 WOS:000313551500006. 23505408 PMC3541386

[33] Davies CE, Moss D, Hill MO. EUNIS habitat classification, revised 2004. Report to the European Topic Centre on Nature Protection and Biodiversity. European Environment Agency; European Topic Centre on Nature Protection and Biodiversity, 2004.

[34] Galparsoro I, Connor DW, Borja A, Aish A, Amorim P, Bajjouk T, et al Using EUNIS habitat classification for benthic mapping in European seas: Present concerns and future needs. Mar Pollut Bull. 2012;64(12):2630–8. 10.1016/j.marpolbul.2012.10.010 WOS:000313380800016. 23117202

[35] Mount R, Bricher P, Newton J. National Intertidal/Subtidal Benthic (NISB) Habitat Classification Scheme. Hobart, Australia: Australian Greenhouse Office; National Land & WaterResources Audit; School of Geography and Environmental Studies, University of Tasmania, 2007.

[36] CMECS Team. Coastal and Marine Ecological Classification Standard. Marine and Coastal Spatial Data Subcommittee of the Federal Geographic Data Committee (FGDC), 2012 FGDC-STD-018-2012.

[37] Mees J, Boxshall GA, Costello MJ, Hernandez F, Gofas S, Hoeksema BW, et al. World Register of Marine Species (WoRMS). 2014.

[38] Howell KL, Davies JS. Deep-Sea Species Image Catalogue Plymouth: Marine Biology and Ecology Research Centre, Marine Institute at the University of Plymouth; 2010 [23/10/2014]. Available: http://www.marlin.ac.uk/deep-sea-species-image-catalogue/.

[39] Przeslawski R, Dundas K, Radke L, Anderson TJ. Deep-sea lebensspuren of the Australian continental margins. Deep-Sea Res Part I-Oceanogr Res Pap. 2012;65:26–35. 10.1016/j.dsr.2012.03.006 WOS:000305862900003.

[40] Reef Life Survey. Reef Life Survey Methods Manual Hobart 2013 [27/11/2014]. Available: http://reeflifesurvey.com/files/2008/09/NEW-Methods-Manual_15042013.pdf.

[41] Schlacher TA, Williams A, Althaus F, Schlacher-Hoenliger MA. High-resolution seabed imagery as a tool for biodiversity conservation planning on continental margins. Mar Ecol. 2010;31:200–21. 10.1111/j.1439-0485.2009.00286.x

[42] Beijbom O, Edmunds PJ, Roelfsema C, Smith J, Kline DI, Neal BP, et al Towards automated annotation of benthic survey images: Variability of human experts and operational modes of automation. PloS ONE. 2015;10(7):e0130312 10.1371/journal.pone.0130312 26154157 PMC4496057

[43] Friedman A. Automated interpretation of benthic stereo imagery [PhD]. Sydney, Australia: The University of Sydney; 2013.

[44] Pizarro O, Williams SB, Jakuba MV, Johnson-Roberson M, Mahon I, Bryson M, et al. Benthic monitoring with robotic platforms—the experience of Australia. 2013 IEEE International Underwater Technology Symposium2013.

[45] Schoening T, Bergmann M, Ontrup J, Taylor J, Dannheim J, Gutt J, et al Semi-automated image analysis for the assessment of megafaunal densities at the Arctic deep-sea observatory HAUSGARTEN. PloS ONE. 2012;7(6):e38179 10.1371/journal.pone.0038179 22719868 PMC3367988

[46] CATAMI. CATAMI Australia2012 [10/08/2015]. Available: http://www.catami.org/.

[47] CATAMI Technical Working Group. CATAMI classification scheme for scoring marine biota and substrata in underwater imagery—Technical Report 2014 [27/11/2014]. Available: http://catami.org/classification] Version 1.4.

[48] Gomes-Pereira JN, Auger V, Beisiegel K, Benjamin R, Bergman M, Bowden D, et al Underwater image annotation software. Progress in Oceanography. in review.

[49] Tran M, Anderson TJ, Booth D, Althaus F, Przeslawski R, Ferrari R, et al. A review of scoring methods for underwater video and still imagery—Presentation given at the Marine imaging Workshop, Southampton 7–10 April. Available: http://www.marine-imaging-workshop.com/documents/MIW14-Program.pdf. 2014.

[50] Neptune Canada. Marine Life Field Guide. Victoria BC: University of Victoria, 2012.

[51] HURL. Animal Identification Guide: Hawai'i Undersea Research Laboratory (HURL); 2013 [updated 26/6/2013; cited 2015 14/7/2015]. Available: http://www.soest.hawaii.edu/HURL/animals/id/.

[52] Rees AJJ, Yearsley, G. K., Gowlett-Holmes, K., and Pogonoski, J. (Codes for Australian Aquatic Biota (on-line version): CSIRO Marine and Atmospheric Research, World Wide Web electronic publication, 1999 onwards; 2014 [27/11/2014]. Available: http://www.cmar.csiro.au/caab/.

[53] Althaus F, Hill N, Edwards L, Ferrari R, Case M, Colquhoun J, et al. CATAMI Classification Scheme for scoring marine biota and substrata in underwater imagery—A pictorial guide to the Collaborative and Annotation Tools for Analysis of Marine Imagery and Video (CATAMI) classification scheme. Version 1.4. Hobart: CSIRO, 2014.

[54] Durnden JM, Shchoening T, Althaus F, Friedman A, Garcia R, Glover A, et al. Perspectives in visual imaging for marine biology and ecology: from acquisition to understanding. Oceanography and Marine Biology: Annual Review. in review.

[55] Hill MS, Hill AL. Morphological plasticity in the tropical sponge *Anthosigmella varians*: Responses to predators and wave energy. Biol Bull. 2002;202(1):86–95. 10.2307/1543225 WOS:000173981800009. 11842018

[56] Loh TL, Lopez-Legentil S, Song B, Pawlik JR. Phenotypic variability in the Caribbean orange icing sponge *Mycale laevis* (Demospongiae: Poecilosclerida). Hydrobiologia. 2012;687(1):205–17. 10.1007/s10750-011-0806-1 WOS:000303066300017.

[57] Meroz-Fine E, Shefer S, Ilan M. Changes in morphology and physiology of an East Mediterranean sponge in different habitats. Mar Biol. 2005;147(1):243–50. 10.1007/s00227-004-1532-2 WOS:000228974100025.

[58] Schönberg CHL, Barthel D. Inorganic skeleton of the demosponge *Halichondria panicea*. Seasonality in spicule production in the Baltic Sea. Mar Biol. 1997;130(2):133–40. 10.1007/s002270050232 WOS:000071438600001.

[59] Sommer B, Harrison PL, Beger M, Pandolfi JM. Trait-mediated environmental filtering drives assembly at biogeographic transition zones. Ecology. 2014;95(4):1000–9. 10.1890/13-1445.1 WOS:000334573600018. 24933818

[60] Schönberg CHL, Fromont J. Sponge functional growth forms as a means for classifying sponges without taxonomy. Available: http://ningaloo-atlas.org.au/ AIMS; 2014 [02/08/2015]. Available: http://ningaloo-atlas.org.au/content/sponge-functional-growth-forms-means-classifying-spo.

[61] Abdo D, Burgess S, Coleman G, Osborne K. Surveys of benthic reef communities using underwater video. Long-term monitoring of the Great Barrier Reef Standard operational procedure 2. 3rd revised edition Townsville: Australian Institute of Marine Science, 2004.

[62] Kohler KE, Gill SM. Coral Point Count with Excel extensions (CPCe): A Visual Basic program for the determination of coral and substrate coverage using random point count methodology. Comput Geosci. 2006;32(9):1259–69. 10.1016/j.cageo.2005.11.009 WOS:000240760200003.

[63] Bax NJ, Hedge P. Marine Biodiversity Hub, National Environmental Research Program, Final report 2011–2015. Report to Department of the Environment. Canberra, Australia: NERP Marine biodiversity Hub, 2015.

[64] NSW Government. Marine Parks 2015 [10/08/2015]. Available: http://www.mpa.nsw.gov.au/research.html).

[65] Chevron. Wheatstone Project dredging and dredge spoil placement environmental monitoring and management plan. Chevron Australia Pty Ltd, 2013 Contract No.: WS0-0000-HES-RPT-CVX-000-00143-000.

[66] Hill NA, Lawrence E, Dambacher JM, Barrett N, Hulls J, Williams A, et al., editors. Developing long-term monitoring programs in offshore waters with little prior knowledge: Applying a novel sampling design to inventory biological assets. Australian Marine Science Association Conference, 7–11 July 2013; 2013; Gold Coast, Australia: AMSA.

[67] Hayes KR, Dambacher J, Hedge P, Watts D, Dunstan P, Bax N. Blueprint for monitoring Key Ecological Features in the Commonwealth marine area. Hobart, Australia: NERP Marine Biodiversity Hub, 2015.

[68] Reef Life Survey. Reef Life Survey 2015 [10/08/2015]. Available: http://reeflifesurvey.com/.

[69] Gerovasileiou V, Voultsiadou E. Sponge diversity gradients in marine caves of the eastern Mediterranean. J Mar Biol Assoc UK. 2015:1–10.

[70] Friedman A. Squidle 2015 [10/08/2015]. Available: http://squidle.acfr.usyd.edu.au/.

[71] CORALNET. CORALNET alpha—A web solution for coral reef analysis 2015 [10/08/2015]. Available: http://coralnet.ucsd.edu/.

[72] Schönberg CHL, Fromont J. Sponge gardens of Ningaloo Reef (Carnarvon Shelf, Western Australia) are biodiversity hotspots. Hydrobiologia. 2012;687(1):143–61. 10.1007/s10750-011-0863-5 WOS:000303066300013.

[73] Tittensor DP, Baco-Taylor AR, Brewin P, Clark MR, Consalvey M, Hall-Spencer J, et al Predicting global habitat suitability for stony corals on seamounts. J Biogeogr. 2009;36(6):1111–28.

[74] Spalding MD, Fox HE, Halpern BS, McManus MA, Molnar J, Allen GR, et al Marine ecoregions of the world: A bioregionalization of coastal and shelf areas. Bioscience. 2007;57(7):573–83. 10.1641/b570707 WOS:000248201700007.

[75] Last PR, Lyne VD, Williams A, Davies CR, Butler AJ, Yearsley GK. A hierarchical framework for classifying seabed biodiversity with application to planning and managing Australia’s marine biological resources. Biological Conservation. 2010;143:1675–86.

[76] Last P, Lyne V, Yearsley G, Gledhill D, Gomon M, Rees T, et al. Validation of national demersal fish datasets for the regionalisation of the Australian continental slope and outer shelf (>40 m depth). Department of the Environment and Heritage and CSIRO Marnine Research, Research DotEaHaCM; 2005.

[77] O'Hara TD, Rowden AA, Bax NJ. A southern hemisphere bathyal fauna is distributed in latitudinal bands. Curr Biol. 2011;21(3):226–30. 10.1016/j.cub.2011.01.002 WOS:000287259600025. 21256017

[78] IMOS. Integrated Marine Oceans Observing System Australia2015 [10/08/2015]. Available: http://imos.org.au/.

[79] CSIRO-MNF. Marine National Facility Australia: CSIRO; 2015 [10/08/2015]. Available: http://mnf.csiro.au/.

[80] AODN. Australian Ocean Data Network Portal Australia: IMOS; 2015 [10/08/2015]. Available: http://portal.aodn.org.au/aodn/.

[81] ANDS. Australian National Data Service Australia2015 [10/08/2015]. Available: http://www.ands.org.au/index.html.

[82] OBIS. Ocean Biogeographic Information System 2015 [10/08/2015]. Available: http://www.iobis.org/.

[83] Jones DOB, Gates AR, Curry RA, Thomson M, Pile A, Benfield M. SERPENT project. Media database archive 2009 [20/11/2014]. Available: http://archive.serpentproject.com/.

[84] PANGEA. PANGEA—Data Publisher for Earth & Environmental Science Germany2015 [10/08/2015]. Available: http://www.pangaea.de/. 10.1038/s41597-023-02269-x PMC10238520 37268655

[85] Schönberg CHL. Monitoring bioeroding sponges: using rubble, quadrat or intercept surveys? Biol Bull. 2015;228(2):137–55. WOS:000353569700006. 25920717 10.1086/BBLv228n2p137

[86] Wells E, Wilkinson M, Wood P, Scanlan C. The use of macroalgal species richness and composition on intertidal rocky seashores in the assessment of ecological quality under the European Water Framework Directive. Mar Pollut Bull. 2007;55(1–6):151–61. 10.1016/j.marpolbul.2006.08.031 WOS:000244158000014. 17074370

[87] Alcolado PM. Reading the code of coral reef sponge community composition and structure for environmental biomonitoring: some experiences from Cuba In: Custódio MR, Hajdu E, Lôbo-Hajdu G, Muricy G, editors. Porifera research: biodiversity, innovation and sustainability. Rio de Janeiro: Museu Nacional; 2007 p. 3–10.

[88] Orfandis S, Panayotidis P, Stamatis N. Ecological evaluation of transitional and coastal waters: A marine benthic macrophytes-based model. Meditteranean Marine Science. 2001;2:45–65.

[89] Littler MM, Littler DS. The evolution of thallus form and survival strategies. The American Naturalist. 1980;116:25–44.

[90] Dustan P, Halas JC. Changes in the reef-coral community of Carysfort Reef, Key Largo, Florida: 1974 to 1982. Coral Reefs. 1987;6(2):91–106. 10.1007/bf00301378 WOS:A1987K625700005.

[91] Kramer PA. Synthesis of coral reef health indicators for the Western Atlantic: results of the AGRRA program (1997–2000). Atoll Research Bulletin. 2003;496:1–55.

[92] Riegl B. Corals in a non-reef setting in the southern Arabian Gulf (Dubai, UAE): fauna and community structure in response to recurring mass mortality. Coral Reefs. 1999;18(1):63–73. 10.1007/s003380050156 WOS:000080295400010.

[93] Sandin SA, Smith JE, DeMartini EE, Dinsdale EA, Donner SD, Friedlander AM, et al Baselines and degradation of coral reefs in the northern Line Islands. PloS ONE. 2008;3(2):e1548 10.1371/journal.pone.0001548 WOS:000260586500001. 18301734 PMC2244711

[94] Kenchington E, Cogswell A, Lirette C, Murillo-Perez FL. The use of density analyses to delineate sponge grounds and other benthic VMEs from trawl survey data. 2009 Contract No.: 09/6.

[95] Parker S, Tracey D, Mackay E, Mills S, Marriott P, Anderson O, et al. CCAMLR VME Taxa Identification Guide Version 2009 Hobart, Australia: Commission for the Conservation of Antarctic Marine Living Resources; 2009. Available: http://www.ccamlr.org/en/document/publications/vme-taxa-classification-guide.

[96] Tracey D, Parker, S. J., Mackay, E., Anderson, O., and Ramm, K.. Classification guide for potentially vulnerable invertebrate taxa in the SPRFMO Area. South Pacific Regional Fisheries Management Organisation, 2008 SP-08-SWG-DW-03.

[97] Heyward A, Fromont J, Schönberg CHL, Colquhoun J, Radford B, Gomez O. The sponge gardens of Ningaloo Reef, Western Australia. The Open Marine Biology Journal. 2010;4(1):3–11. 10.2174/1874450801004010003

[98] ISO. ISO—Standards 2015 [10/08/2015]. Available: http://www.iso.org/iso/home/standards.htm.

[99] OPG. Offshore environmental monitoring for the oil & gas industry. UK: international Association of Oil & Gas Producers, 2012.

[100] NOPSEMA. National Offshore Petroleum Safety and Environmental Management Authority Australia: Australian Government; 2015 [10/08/2015]. Available: http://www.nopsema.gov.au/.

[101] Seiler J, Friedman A, Steinberg D, Barrett N, Williams A, Holbrook NJ. Image-based continental shelf habitat mapping using novel automated data extraction techniques. Cont Shelf Res. 2012;45:87–97.

[102] Edgar GJ, Stuart-Smith RD. Ecological effects of marine protected areas on rocky reef communities-a continental-scale analysis. Mar Ecol-Prog Ser. 2009;388:51–62. 10.3354/meps08149 WOS:000269892700006.

[103] Stuart-Smith RD, Bates AE, Lefcheck JS, Duffy JE, Baker SC, Thomson RJ, et al Integrating abundance and functional traits reveals new global hotspots of fish diversity. Nature. 2013;501(7468):539-+. 10.1038/nature12529 WOS:000324826300056. 24067714

[104] Andrews K, Ramsden J, Wilson L, R S. Explore the seafloor—ABC Science Week 2013, 2013 [27/11/2014]. Available: www.exploretheseafloor.net.au.

[105] Zooniverse. Seafloor Explore: Zooniverse; 2015 [10/08/2015]. Available: http://www.seafloorexplorer.org.

[106] ABC Science, IMOS. Explore the Seafloor Australia: ABC Science & IMOS; 2015 [10/08/2015]. Available: http://exploretheseafloor.net.au/.

[107] Bird TJ, Bates AE, Lefcheck JS, Hill NA, Thomson RJ, Edgar GJ, et al Statistical solutions for error and bias in global citizen science datasets. Biological Conservation. 2014;173:144–54. 10.1016/j.biocon.2013.07.037 WOS:000336874100017.

[108] Fithian W, Elith J, Hastie T, Keith DA. Bias correction in species distribution models: pooling survey and collection data for multiple species. Methods Ecol Evol. 2014:n/a-n/a. 10.1111/2041-210X.12242 PMC5102514 27840673

[109] Royle JA, Chandler RB, Yackulic C, Nichols JD. Likelihood analysis of species occurrence probability from presence-only data for modelling species distributions. Methods Ecol Evol. 2012;3(3):545–54. 10.1111/j.2041-210X.2011.00182.x WOS:000304902500013.

[110] Warton DI, Renner IW, Ramp D. Model-based control of observer bias for the analysis of presence-only data in ecology. PloS ONE. 2013;8(11). e79168 10.1371/journal.pone.0079168 WOS:000327308500031. 24260167 PMC3832482

[111] Kirk P, Griffin JE, Savage RS, Ghahramani Z, Wild DL. Bayesian correlated clustering to integrate multiple datasets. Bioinformatics. 2012;28(24):3290–7. 10.1093/bioinformatics/bts595 WOS:000312105300018. 23047558 PMC3519452

