# Supplementary material for: A Standardised Vocabulary for Identifying Benthic Biota and Substrata from Underwater Imagery: The CATAMI Classification Scheme
Source: PLoS One. 2015 Oct 28;10(10):e0141039. doi: 10.1371/journal.pone.0141039 (PMC4625050; doi:10.1371/journal.pone.0141039)
Supplement: S1 Appendix — (DOCX) [file pone.0141039.s001.docx]

### S1 Appendix — CATAMI Project: Initial stakeholder workshop – aims, participants & outcomes

The CATAMI Project, funded through the Australian National Data Service (ANDS) and NeCTAR, sought to improve the efficiency of labelling underwater imagery and enhance its management. The project’s aim was to establish a framework applicable to marine imagery that will facilitate the timely delivery of standardised, quantitative estimates of ecologically relevant indicators (such as absence/presence, percent cover, abundance and distribution of benthic organisms and associated substrates) through the analysis of visual data being produced by Autonomous Underwater Vehicles (AUV), Baited Remote Underwater Video Systems (BRUVS), Remotely Operated Vehicles (ROV) and Underwater Towed Video (UTV) systems.

An initial marine imagery stakeholder workshop, organized by A/Prof Stefan Williams (Australian Centre for Field Robotics - University of Sydney), was held on 26^th^ and 27^th^ March 2012 at the Sydney Institute of Marine Science (SIMS), Chowder Bay, New South Wales, bringing together Australian researchers in the field of marine ecology and habitat monitoring. Attendees (listed in Table 1) gave short presentations of the image data collections, annotation systems and image analyses tools specific to their research areas and institutions. This was followed by discussion regarding the requirements for making image data from various fields of research discoverable and accessible, in order to maximize the use of this resource; in addition, requirements for automated classification algorithms were discussed.

The need to develop an Australian wide standardised classification scheme for identifying biota and substrata in underwater imagery was identified as a critical aspect to achieve this goal.

**S1-Table 1 List of attendees at the CATAMI Project initial stakeholder workshop**

A/Prof Stefan Williams University of Sydney

Donald Dansereau University of Sydney

Dr Navid Nourani-Vatani University of Sydney

Dr Oscar Pizarro University of Sydney

Dr Bertrand Douillard University of Sydney

Daniel Steinberg University of Sydney

Mr Michael Bewley University of Sydney

Dr Mitchell Bryson University of Sydney

Prof Maria Byrne University of Sydney

Dr Matthew Johnson-Roberson University of Sydney

Luke Edwards Pawsey Supercomputing Centre

Prof Jenni Harrison Pawsey Supercomputing Centre

Mathew Wyatt Pawsey Supercomputing Centre

Dr Keith Hayes CSIRO - NERP

Dr Russell Babcock CSIRO, Cleveland

Dr Pamela Brodie CSIRO, Hobart

Franziska Althaus CSIRO, Hobart

Mr Laurent Besnard eMII, IMOS

Dr Roger Proctor eMII, IMOS

Mark Case AIMS

Marcus Stowar AIMS

Jonathan Kool Geoscience Australia

Maggie Tran Geoscience Australia

Andrew Carroll Geoscience Australia

Alix Post Geoscience Australia

Dr Rachel Przeslawski Geoscience Australia

Dr Alan Jordan NSW Government

Dr Tom Bridge James Cook University

Dr Andy Davis University of Wollongong

Dr Nicole Hill University of Tasmania

Dr Martin Marzloff University of Tasmania

Dr Ezequiel Marzinelli University of New South Wales

Prof Gary Kendrick University of Western Australia

Eric Grossman University of Western Australia

A/Prof Euan Harvey University of Western Australia
