# Supplementary material for: A Standardised Vocabulary for Identifying Benthic Biota and Substrata from Underwater Imagery: The CATAMI Classification Scheme
Source: PLoS One. 2015 Oct 28;10(10):e0141039. doi: 10.1371/journal.pone.0141039 (PMC4625050; doi:10.1371/journal.pone.0141039)
Supplement: S2 Appendix — (DOCX) [file pone.0141039.s002.docx]

### S2 Appendix ­— List of the 19 members of the CATAMI Technical Working Group and additional contributors to the CATAMI classification scheme (CCS).

| **Institution** | **Acronym** | **Key contributors** | **Image data research focus** | **Classification branch adopted in CCS** |
| --- | --- | --- | --- | --- |
| Pawsey Supercomputing Centre | Pawsey | Luke Edwards | Data management; CATAMI Project coordination |  |
| Australian Institute of Marine Science | AIMS | Jamie Colquhoun,  Mark Case | Coral reef environments | Stony corals  Shallow octocorals |
| Commonwealth Scientific and Industrial Research Organisation | CSIRO | Franziska Althaus,  Karen Gowlett-Holmes* | Outer shelf, continental slope and seamounts | Octocorals/Black corals  Bryozoa |
|  |  | Tony Rees | CAAB administration |  |
|  |  | Keith Hayes | Monitoring of reserves |  |
| Geoscience Australia | GA | Rachel Przeslawski,  Scott Nichol* | Geological environments and sediment compositions | Physical classification |
|  |  | Rachel Przeslawski,  Maggie Tran* | Lebenspuren in deep sediments | Bioturbation  [1] |
| Australian Antarctic Division | AAD | Ty Hibberd | Antarctic habitats |  |
| NSW Department of Industries | NSW DPI | Alan Jordan | Subtropical and temperate inner shelf benthic habitats |  |
| University of Western Australia, Western Australian Museum | UWA, WAM | Christine Schönberg | Sponges – functional morphology | Sponges  [2] |
| Western Australian Museum | WAM | Jane Fromont | Sponges – functional morphology | Sponges  [2] |
| Institute for Marine and Antarctic Studies - University of Tasmania | IMAS/ UTAS | Nicole Hill,  Graham Edgar,  Fiona Scott,  Neville Barrett* | Temperate inner shelf  Temperate cross shelf | Macroalgae  Seagrasses |
| Australian Centre for Field Robotics and School of Biological Sciences - University of Sydney | ACFR &Sydney University | Renata Ferrari | Temperate, sub-tropical and tropical inner shelf habitats | Stony corals  Shallow octocorals |
| Centre for Field Robotics - University of Sydney | ACFR | Ariell Friedmann  Daniel Steinberg | Automation of image annotation |  |
|  |  | Lachlan Toohey | Robotics (AUV) |  |
| Sydney Institute of Marine Science | SIMS | Ezequiel Marzinelli | Kelp distributions |  |
| University of Western Australia | UWA | Renae Hovey, Gary Kendrick | Kelp and seagrass distributions  Temperate and sub-tropical inner shelf habitats |  |

The respective research focus with regard to imagery and their affiliation are shown for each contributor; contributors additional to the working group are indicated with an asterisk. Specific branches of the CCS that were adopted from a particular institution’s existing classification are identified in the last column.

**References**

1. Przeslawski R, Dundas K, Radke L, Anderson TJ. Deep-sea lebensspuren of the Australian continental margins. Deep-Sea Res Part I-Oceanogr Res Pap. 2012;65:26-35. doi: 10.1016/j.dsr.2012.03.006. PubMed PMID: WOS:000305862900003.

2. Schönberg CHL, Fromont J. Sponge functional growth forms as a means for classifying sponges without taxonomy <http://ningaloo-atlas.org.au/:> AIMS; 2014 [02/08/2015]. Available from: <http://ningaloo-atlas.org.au/content/sponge-functional-growth-forms-means-classifying-spo>.
