# Supplementary material for: A Standardised Vocabulary for Identifying Benthic Biota and Substrata from Underwater Imagery: The CATAMI Classification Scheme
Source: PLoS One. 2015 Oct 28;10(10):e0141039. doi: 10.1371/journal.pone.0141039 (PMC4625050; doi:10.1371/journal.pone.0141039)
Supplement: S3 Appendix — (DOCX) [file pone.0141039.s003.docx]

### S3 Appendix — Meetings where the CATAMI Classification was presented and discussed

- Australian Marine Science Association Conference, July 2012
- Seminar given at the Australian Government Department of Environment and Conservation (now Dept. of Parks and Wildlife), October 2012
- National Environmental Research Program (NERP) Symposium, November 2012
- University of Western Australia – Big Data Week, April 2013
- Marine Geological and Biological Habitat Mapping Conference, Rome (I), May 2013
- Australian Marine Science Association Conference, July 2013
- AeRO Workshop, July 2013
- Australian Coral Reef Society, August 2013
- eResearch Australasia Conference, October 2013
- 9^th^ World Sponge Conference, November 2013
- WALIS Forum conference, November 2013
- Marine Imaging Workshop, Southampton (UK), April 2014
- Australian Marine Science Association Conference, July 2014
- WAiTTA presentations and INCITE award within the Society Domain, Sustainability category (<http://iawards.com.au/index.php/winners/2014-winners/2014-winners-waitta>) 2014
- APPEA HSE Conference, September 2015
